# HHLA2 predicts improved prognosis of anti-PD-1/PD-L1 immunotherapy in patients with melanoma

**DOI:** 10.3389/fimmu.2022.902167

**Published:** 2022-08-08

**Authors:** Fu-xue Huang, Jun-wan Wu, Xia-qin Cheng, Jiu-hong Wang, Xi-zhi Wen, Jing-jing Li, Qiong Zhang, Hang Jiang, Qiu-yue Ding, Xiao-feng Zhu, Xiao-shi Zhang, Ya Ding, Dan-dan Li

**Affiliations:** ^1^ Biotherapy Center, State Key Laboratory of Oncology in South China, Collaborative Innovation Center for Cancer Medicine, Sun Yat-sen University Cancer Center, Guangzhou, China; ^2^ State Key Laboratory of Oncology in South China, Collaborative Innovation Center for Cancer Medicine, Sun Yat-sen University Cancer Center, Guangzhou, China; ^3^ Department of Radiation Oncology & Therapy, Jilin Provincial Key Laboratory of Radiation Oncology & Therapy, The First Hospital of Jilin University, Changchun, China; ^4^ Department of Gynecological Oncology, Sun Yat-Sen University Cancer Center, Guangzhou, China; ^5^ Department of Radiotherapy, Affiliated Yantai Yuhuangding Hospital of Qingdao University, Yantai, China

**Keywords:** melanoma, HHLA2, immunotherapy, prognosis, tumor infiltrating lymphocytes

## Abstract

**Background:**

As a recognized highly immunogenic tumor, immune checkpoint blockades (ICB) have been widely used as a systemic treatment option for melanoma. However, only about half of treated patients could benefit from it in Caucasians, and only about 15% in Chinese melanoma patients. Robust predictive biomarkers are needed. HHLA2, a new-found member of B7 family, is generally expressed in kinds of tumors, such as melanoma. This study focuses on illustrating the prognostic value of HHLA2 in melanoma immunotherapy and its association with tumor-infiltrating lymphocytes.

**Methods:**

HHLA2 expression in pan-cancer and the association with prognosis and immune microenvironment were identified by analyzing gene expression profiles from TCGA database with selected bioinformatics tools and methods. Tumor tissues from 81 cases with advanced and unresectable melanoma were collected for detecting HHLA2 and CD8 levels by immunohistochemistry.

**Results:**

HHLA2 was found to be ubiquitously expressed in pan-cancer with high level and correlate with the prognosis of patients. Further comprehensive analysis from TCGA database demonstrated that the highly expressed HHLA2 was remarkably correlated with better prognosis, high infiltration status of various immune-active cells and immune activated pathways in skin cutaneous melanoma (SKCM). Moreover, immunohistochemistry (IHC) analyses of FFPE tissue from melanoma patients revealed that HHLA2 high expression was strongly related to improved response to ICB and indicated a longer progression-free survival (PFS) and overall survival (OS). Besides, HHLA2 expression was found to have a positive association with the density of CD8^+^ TILs.

**Conclusion:**

Our findings revealed that high expression of HHLA2 has important values in predicting the response to ICB and indicating improved PFS and OS in patients with advanced and unresectable melanoma, suggesting that HHLA2 may serve as a costimulatory ligand in melanoma, which renders it as an ideal biomarker for immunotherapy.

## Introduction

Melanoma is a lethal skin cancer derived from melanocytes. Despite melanoma often occurs in skin areas which are exposed to the sun, it can also occur to the eyes and trunk where sunlight exposure is avoided (1). Melanoma accounts for only about 1% among all cutaneous cancer, which is much less common compared to other kinds of cutaneous cancer. Over the past few decades, melanoma has becoming more and more common with increasing incidence all over the world (2). The American Cancer Society has claimed that about 106,110 melanomas cases were newly diagnosed in the US in 2021 (31.91 per 100,000 person-years) (3). As reported by the Global Burden of Disease Study in 2017, in China, the age-standardized incidence of melanoma was 0.9 per 100,000 person-years (4). Despite its rareness, melanoma is recognized as the primary reason for skin cancer-related death (3).

In the last few years, the development of immune checkpoint blockades (ICBs) has had great influence on the clinical treatment of advanced melanoma. Melanoma, being the first indication for nivolumab and pembrolizumab which were approved in 2014 (5, 6), is suggested as one of the immunogenic cancer types which possesses fine response to PD-1 blockade. ICBs have emerged as a first-line treatment for melanoma due to their potential ongoing clinical efficacy and advantages in benefiting patient survival. The 9-month Median overall survival (mOS) of metastatic melanoma treated by dacarbazine chemotherapy was improved to 6.5 years when treated by nivolumab plus ipilimumab in the CheckMate 067 trial, and potential long-term benefits after withdrawal was observed (7). Although ICB has considerable therapeutic promise, there are about only a half of patients who are therapy-responsive, not to mention the primary or acquired ICB resistance which have been observed for many patients (8, 9). In addition, ICBs bring the risk of severe immune-related side effect which may cause death (10). To avoid such events, patients who well respond to ICBs and gain benefits from this therapy could be identified by specific biomarkers, including PD-L1 expression, tumor mutation burden (TMB), microsatellite instability (MSI), and interferon γ (IFN-γ) gene signatures (11, 12). However, the predictive value of PD-L1 expression is limited because of the heterogeneity among patients. Therefore, further study of tumor microenvironment (TME) has emphasized its potential and essential roles in revealing new and reliable biomarkers.

HHLA2, short for HERV-HLTR-associating 2, also known as B7H7/B7-H5/B7y, is a specific molecule in TME which has drawn our attention (13, 14). HHLA2 is a newly discovered molecule to ubiquitously and highly expressed in many cancers and belongs to the B7 family (15–20). HHLA2, as a type I transmembrane molecule, contains three extracellular Ig domains, and its receptors are distributed throughout a variety of immune cells, such as T cells, monocytes, B cells, and endothelial cells (21), two of which have been identified, including TMIGD2 which belongs to the CD28 family and is mainly expressed on naive T cells and natural killer cells (22, 23), KIR3DL3 which is mainly expressed on terminal differentiation effector memory CD8^+^ T cells and CD56^dim^ CD16^+^ NK cells (24). In conclusion, up-regulated HHLA2 level indicates severer pathology and worse prognosis for different cancers, such as clear cell renal cell carcinoma (ccRCC), intrahepatic cholangiocarcinoma (ICC) and osteosarcoma (15–17). However, some studies have suggested that high HHLA2 expression is an independent predictor for better survival of pancreatic ductal adenocarcinoma and ccRCC (18–20).

By far as we acknowledge, there has not any study focusing on clarifying the relationship between the expression of HHLA2 and the prognosis of melanoma patients treated by immunotherapy that has been reported to date. Meanwhile, the relationship between HHLA2 and tumor immune microenvironment of melanoma has yet been explored.

In this study, we mainly aimed to figure out the relationship between HHLA2 expression and clinicopathological characteristics, prognosis and immune infiltrations of melanoma. As a result, analyses revealed that increased HHLA2 expression was associated with better ICB response and increased infiltration of CD8^+^ T cells in the cohort study of Asian patients diagnosed with advanced or unresectable melanoma. This study may provide practical guidance of more accurate confirmation for melanoma patients who may benefit from immunotherapy.

## Materials and methods

### Patients and tumors

There was a whole of 81 archived specimens fixed by formalin and embedded in paraffin (FFPE) specimens, which were collected from Asian patients with advanced or unresectable melanoma who received first line or second line ICB therapy during October 2014 and December 2021 at the biotherapy center of Sun Yat-Sen university cancer center and evaluated. These samples were obtained from surgical resections or biopsy samples before immunotherapy. Clinicopathological parameters are summarized in **Table 1**.

**Table 1:** Clinicopathological characteristics of patients enrolled in our cohort.

Characteristics	HHLA2 Low N=63	HHLA2 High N=18	P value
**Age**			0.155
<60	49 (81.7)	11 (18.3)	
≥60	14 (66.7)	7 (33.3)	
**Gender**			0.282
Male	37 (82.2)	8 (17.8)	
Female	26 (72.2)	10 (27.8)	
**ECOG PS**			0.676
0	55 (76.4)	17 (23.6)	
1-2	8 (88.9)	1 (11.1)	
**LDH**			0.724
Normal	51 (76.1)	16 (23.9)	
Elevated	12 (85.7)	2 (14.3)	
**Subtype**			0.424
Cutaneous	30 (76.9)	9 (23.1)	
Acral	21 (72.4)	8 (27.6)	
Mucosal	12 (92.3)	1 (7.7)	
**Tumor stage**			1.000
Stage III	4 (80.0)	1 (20.0)	
Stage IV	59 (77.6)	17 (22.4)	
**Liver metastasis**			0.175
No	50 (74.6)	17 (25.4)	
Yes	13 (92.9)	1 (1.2)	
**Brain metastasis**			0.677
No	56 (76.7)	17 (23.3)	
Yes	7 (87.5)	1 (12.5)	
**Line of systematic treatment**			0.538
First	46 (75.4)	15 (85.0)	
Second	17 (24.6)	3 (15.0)	
**Type of immunotherapy**			0.548
Anti-PD-1/PD-L1/CTLA4 monotherapy	23 (76.7)	7 (23.3)	
Anti-PD-1/PD-L1+ Anti-CTLA-4 therapy	6 (66.7)	3 (33.3)	
Anti-PD-1/PD-L1+ other therapies	34 (81.0)	8 (19.0)	

*P values <0.05 in bold are statistically significant.

HHLA2, human endogenous retrovirus-H long terminal repeat-associating protein 2; ECOG PS, Eastern Cooperative Oncology Group Performance Status; LDH, lactate dehydrogenases; PD-1, programmed cell death protein-1; PD-L1, programmed death 1 ligand 1; CTLA-4, cytotoxic T-lymphocyte-associated protein 4.

A total of 81 patients (36 females and 45 males, overall median age 53 years) were included in this analysis (cutaneous n=39, acral n=29, and mucosal n=13). 24.7% (20/81) of patients received more than one line of treatment. 37.0% (30/81) of patients received anti-PD-1/PD-L1/CTLA4 monotherapy and 11.1% (9/81) received anti-PD-1/PD-L1 combined with anti-CTLA-4 therapy. In patients receiving anti-PD-1/PD-L1 combined with other therapies (51.9%, 42/81), there were 5 combined with chemotherapy and 3 combined with targeted therapy in high HHLA2 expression group, and 23 combined with chemotherapy, 11 combined with targeted therapy, 2 combined with anti-angiogenic drugs in low HHLA2 expression group.

### Immunohistochemistry

Immunohistochemistry (IHC) staining of HHLA2 and CD8 was carried out by a professional pathologist. Samples were processed through deparaffinization, rehydration, antigen retrieval, endogenous peroxidase inactivation, and blockage of non-specific binding, and incubated with anti-HHLA2 and anti-CD8 primary antibodies (anti-HHLA2: Sigma-Aldrich, HPA055478; anti-CD8: CST, #85336) at 4°C overnight, after which the slides were then co-incubated with secondary antibodies and colored by 3,30-diaminobenzidine. Then sections were counterstained by hematoxylin, rinsed in running tap water, followed by dehydration, and microscope was applied to observe the results. Samples treated without primary antibodies were set as controls.

### Quantification of HHLA2 and infiltration of T cells

Slides were independently observed and evaluated by 2 investigators under the guidance of a professional pathologist without referring to the corresponding clinical profiles. Discrepant results between two investigators were discussed and re-evaluated together.

The results of HHLA2 staining were assessed and scored according to the proportion of positive staining of tumor cells. “Low expression” was set as <50% of HHLA2 staining, while proportion > 50% was considered as “high expression”. CD8 ^+^ cells were counted under ×400 magnification in at least five randomly-chosen high-power fields, and the CD8^+^ T-cell density was calculated according to the average number of positively stained cells.

### Gene and pathway analysis

Gene expression profile and clinical information were acquired from The Cancer Genome Atlas (TCGA; https://www.cancer.gov/tcga). Tumor samples were distinguished by ‘high’ or ‘low’ HHLA2 expression based on an optimal threshold that resulted in the lowest p value. Differential expression of mRNA was analyzed by the Limma package of R software (version 4.1.2, R-core Team, R Foundation for Statistical Computing, Vienna, Austria). Adjusted p values were analyzed for correcting false-positive results. DEGs were accessed by screening under the conditions of |log2(FC)| > 2, P< 0.05. The ClusterProfiler package in R was applied to analyze the GO function of potential mRNAs and enrich the KEGG pathway.

### Gene set enrichment analysis and immunological association analysis

HALLMARK pathways in the high and low expression group were respectively analyzed by GSEA. The associations between gene expression and 22 immune cells infiltration were analyzed by CIBERSORT. The immune scores and stromal scores of each sample were evaluated using the R package ESTIMATE.

### Statistical analysis

Patient follow-up data were acquired from medical records. The χ2 test and Fisher’s exact tests were applied to analyze the association, whereas the Kaplan-Meier method was utilized to conduct survival analysis, by which the survival curves of progression-free survival (PFS) and overall survival (OS) were plotted. OS was defined as the time from the beginning of treatment to death or to the date of the last follow-up. PFS represented the period starting from the first day of treatment to either death, progression, or the date of the last follow-up. Univariate and multivariate analyses were carried out by Cox proportional hazard model. All statistical analyses were accomplished by SPSS V.20.0 software and Graphpad Prism 7 software (La Jolla, California, USA). P value <0.05 was considered to be statistically significant.

## Results

### Expression of HHLA2 in SKCM and other cancer

**Figure 1:**
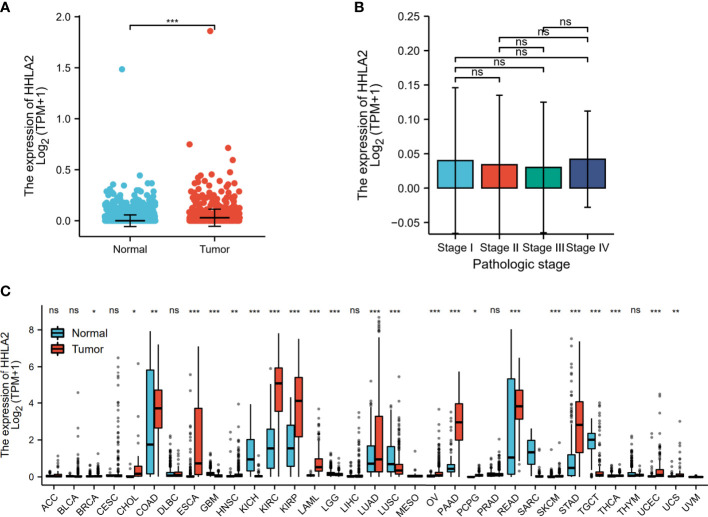
Expression levels of HHLA2 in different tumors; **(A)** Expression level of HHLA2 in SKCM; **(B)** Expression levels of HHLA2 in SKCM at different TNM stages; **(C)** Expression levels of HHLA2 in 33 types of cancers. ‘*’ stands for P < 0.01, ‘**’ stands for P < 0.001, ‘***’ stands for P < 0.0001. Ns, no significance.

As shown in **Figure 1A**, the expression level of HHLA2 in SKCM was examined and exhibited a high expression in cancerous tissues, and data in **Figure 1B** displayed the expression level of HHLA2 in SKCM at different stages. Moreover, data of normal tissues which was obtained from the GTEx database and data of tumor tissues which was obtained from TCGA were integrated for analyzing the differences in HHLA2 expression levels among 33 tumor types. The results demonstrated high HHLA2 expressions in 17 kinds of cancers, such as COAD, KIRC, SKCM, PAAD et al., and low expressions in 6 cancers, such as GBM and KICH et al. (**Figure 1C**).

### Prognostic analysis of HHLA2 expression in SKCM and other cancers

**Figure 2:**
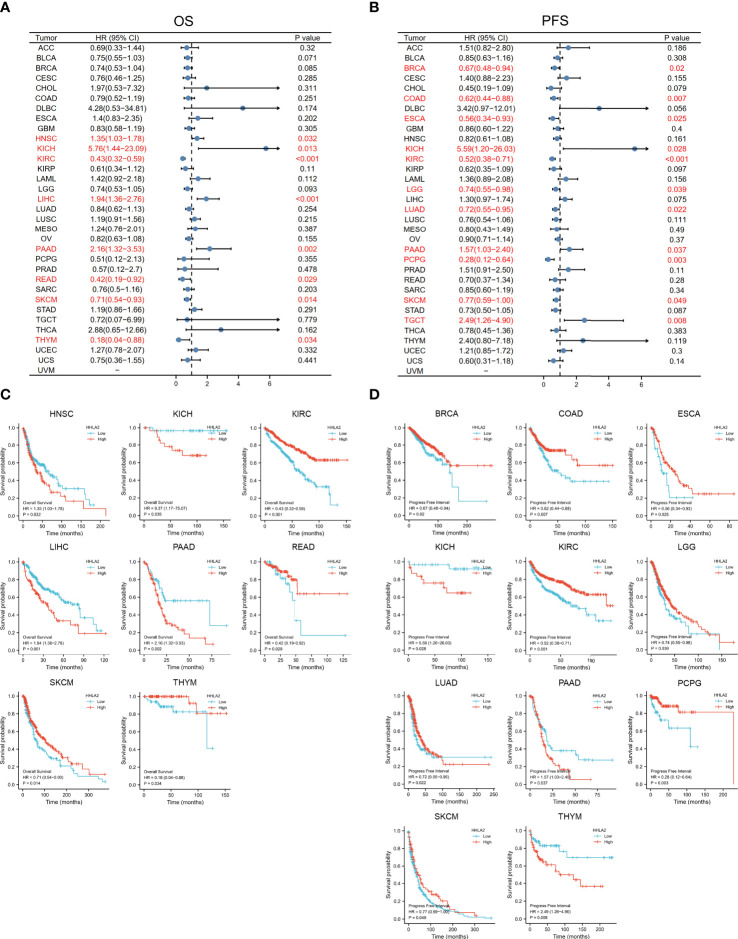
The relationship between HHLA2 expression and survival time of 33 kind of tumors was analyzed by univariate survival analysis. **(A)** Forest plot exhibited the relationship between HHLA2 expression and OS; **(B)** The relationship between HHLA2 expression and PFS; **(C)** KM curves of high and low HHLA2 expression in 8 tumors demonstrated the significant association between HHLA2 expression level and OS survival; **(D)** KM curves of high and low HHLA2 expression in 11 tumors indicated that HHLA2 was significantly associated with PFS.

The associations between HHLA2 expression and overall survival or progression-free survival in 33 TCGA tumors were analyzed using univariate survival analysis. As shown in **Figure 2A**, the overall survival of HNSC, KICH, KIRC, LIHC, PAAD, READ, SKCM and THYM accordingly fluctuated along with the varied expression levels of HHLA2. The Kaplan-Meier curves in **Figure 2B** indicated that, HHLA2 expression level was positively correlated with the prognosis of patients with KIRC, READ, SKCM and THYM, but negatively correlated with the OS of patients with HNSC, KICH, LIHC and PAAD. Furthermore, the association between HHLA2 expression and PFS was depicted in **Figures 2C**, **D**, and the results suggested that the PFS of BRCA, COAD, ESCA, KIRC, LGG, LUAD, PCPG and SKCM was significantly longer in patients with higher HHLA2 expression levels, whereas lower HHLA2 expression corresponded to shorter PFS. However, situations were inverse in KICH, PAAD and THYM. Overall, the results hinted us that HHLA2 may be a potential prognostic predictor in SKCM and other cancers.

### Correlation among HHLA2, tumor immune infiltration and tumor microenvironment in SKCM

**Figure 3:**
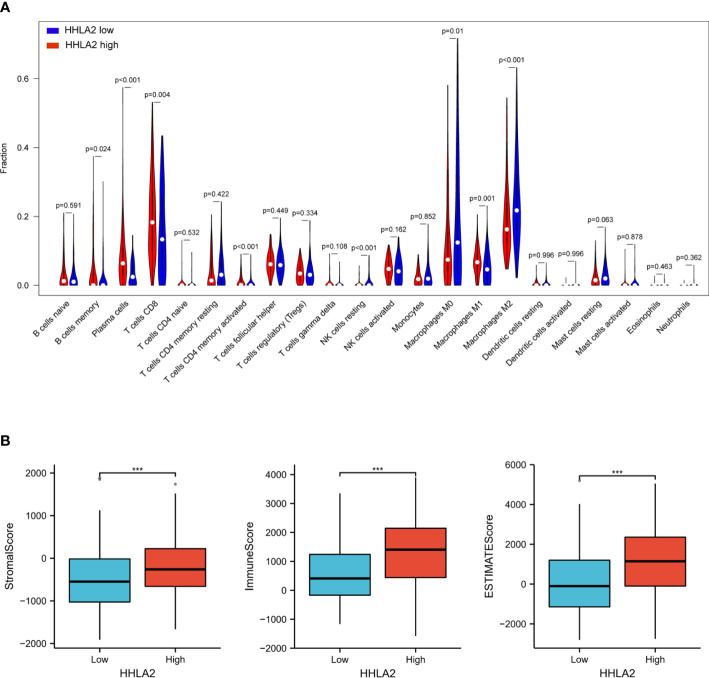
Associations among HHLA2, tumor immune infiltration and the tumor microenvironment in SKCM. **(A)** Varied abundances of 22 infiltrating immune cell types in high and low HHLA2 expression groups. **(B)** Association between high/low HHLA2 expression group and TME score. ***P<0.001. P values <0.05 are in bold.

We then investigated whether HHLA2 expression was correlated with the level of immune infiltration in SKCM. To identify the role of HHLA2 in the TME of SKCM, the associations between the HHLA2 expression and 22 kind of immune cell subtypes in every SKCM sample were analyzed by the CIBERSORT algorithm (**Table S1**).The infiltration of most kind of immune cells was significantly different between the two groups, where the infiltration levels of memory B cells, plasma cells, CD8 + T cells, CD4 memory-activated T cells and M1 macrophages were obviously higher in the HHLA2 high group, but much lower in the HHLA2 low group, while M2 macrophage infiltration level exhibited a contrary association with HHLA2 expression level (**Figure 3A**).

The TME score (stromal score, immune score, and estimate score) of SKCM was assessed by the ESTIMATE package, and its relation with the HHLA2 expression level was also analyzed. For the TME scores, higher stromal score and/or immune score corresponds to higher relative content of stromal cells and/or immunocytes in the TME, while the aggregation of stromal or immune scores in the TME is presented by estimate score. The results demonstrated that patients with HHLA2 high expressions gained higher TME scores (**Figure 3B**).

### GSEA analysis of high and low expression of HHLA2 in SKCM

**Figure 4:**
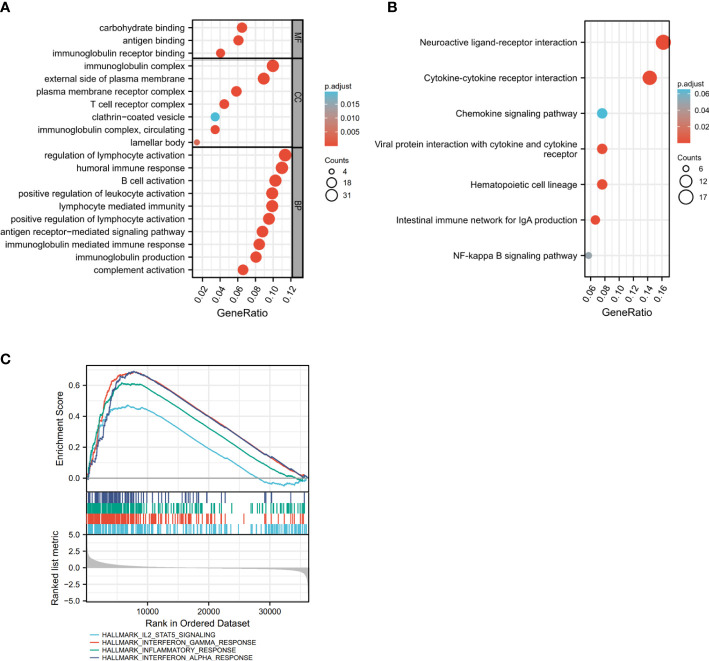
Identification of gene subtypes based on DEGs. **(A, B)** GO and KEGG enrichment analyses of DEGs in high and low HHLA2 expression groups. **(C)** GSEA analysis of HHLA2; enrichment analysis of HHLA2 in HALLMARK signaling pathway. P values <0.05 are in bold.

The GSEA enrichment analysis was conducted to uncover the biological behavior behind HHLA2 expression pattern, and the result suggested that HHLA2 was engaged in the regulation of multiple immune-related cancer biological processes. GO analysis and KEGG implied the enrichment of immune-related signaling pathways, as shown in **Figures 4A**, **B**, indicating that HHLA2 played a key role in the immune regulation of the TME. The analytic result of the interaction between HALLMARK pathway and HHLA2 high expression was displayed in **Figure 4C**, where HHLA2 high expression exhibited positive impacts on IL2-STAT5 signaling, Interferon-gamma response and Interferon-alpha response.

### HHLA2 expression level predicted response to immunotherapy in melanoma

**Figure 5:**
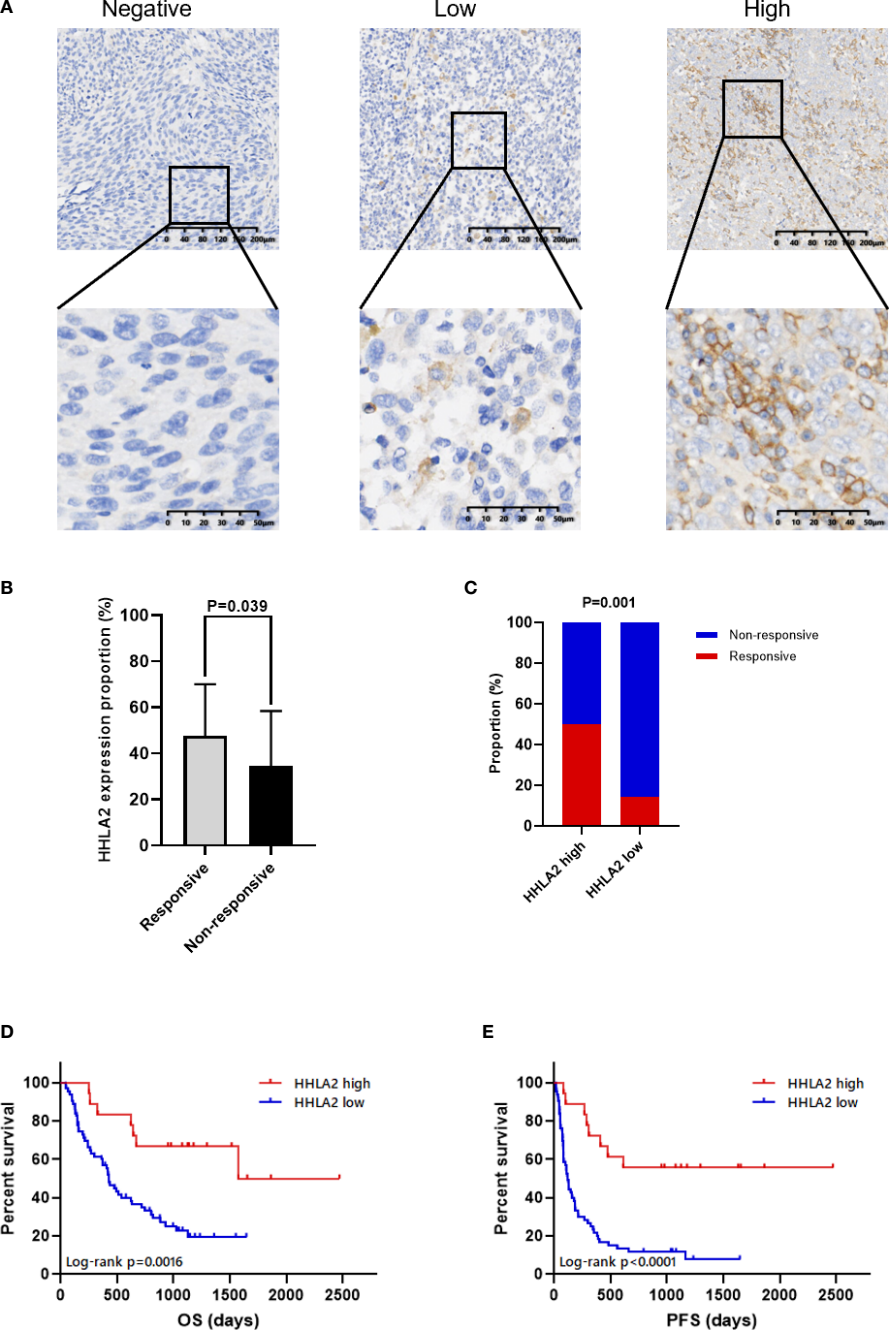
The association between HHLA2 expression and response to ICB in patients with melanoma. **(A)** Representative micrographs of HHLA2 expression in tumor. **(B)** The proportions of HHLA2 expression in tumor tissues from ICB-treated responders and non-responders (unparalleled t-test, p=0.039). **(C)** The response rates of ICB-treated patients in high and low HHLA2 expression group (χ2 test, p=0.001). **(D)** KM curves illustrated that high HHLA2 expression was associated with improved PFS after ICB therapy. **(E)** KM curves showed that high HHLA2 expression was related to improved OS after ICB treatment. P values <0.05 are in bold.

The value of HHLA2 presented on the FFPE tissues of ICB-treated patients with advanced melanoma or unresectable melanoma predicting the response to therapy was evaluated. According to RECIST v.1.1, 18 patients who achieved a complete response (CR) or partial response (PR) were considered as responders, whereas 63 patients who achieved stable disease (SD) or progressive disease (PD) were classified as non-responders. HHLA2 expression was detected at protein level by IHC and scored by traditional manual scoring methods as described in the Materials and Methods section, according to which patients were divided into two groups, including HHLA2-high group and HHLA2-low group. Colocalization experiments by immunofluorescence showed that HHLA2 mainly expressed in melanoma cells, but minorly expressed in CD45+ immune cells (**Figure S1A**). There was no significant difference in HHLA2 expression among different tumor stages and treatment lines (**Figure S2A**–**D**). Responders had significantly higher HHLA2 expression levels than non-responders (**Figure 5A**, **B**, p=0.039). Furthermore, the overall response rate (ORR) was 50% in HHLA2-positive patients and 14.3% in negative patients (**Figure 5C**), suggesting that high HHLA2 expression could be recognized as a sign of better response to immunotherapy in melanoma patients.

### High HHLA2 expression was associated with better prognosis in the immunotherapy cohort

The relationship between HHLA2 expression level and prognosis was analyzed by a univariate Cox proportional hazards regression, which indicated that the risks of disease progression and death were significantly lower in patients with high HHLA2 expression (PFS: HR=0.233, 95%CI: 0.110 to 0.494, p<0.001, **Table 2**; OS: HR=0.290, 95%CI: 0.129 to 0.653, p=0.003, **Table 3**). The result of Kaplan-Meier analysis showed better mPFS and mOS in HHLA2-high patients when compared with HHLA2-low patients, and the median follow-up time was 38.2 months (range 3.5 - 162.4 months). Moreover, mPFS was 31.7 months in HHLA2-high patients, but only 3.9 months in HHLA2-low patients (p<0.0001; **Figure 5D**). Similarly, mOS was 52.4 months in HHLA2-high patients, while it was only 14.2 months in HHLA2-low patients (p=0.0016; **Figure 5E**). Further analysis showed that the high expression of HHLA2 was a good prognostic factor for cutaneous and acral patients receiving immunotherapy. However, statistical significance was not obtained in mucosal melanoma due to the small number of patients. In general, HHLA2 has the same predictive effect in different types of melanoma in our cohort (**Figure S3A**–**F**).

**Table 2:** Univariate analysis of prognostic factors correlated with PFS.

Characteristics	mPFS (months)	HR (95%CI)	P value
**HHLA2 expression**			
Low	3.9	Reference	
High	31.7	0.233 (0.110 to 0.494)	**<0.001**
**Age**			
<60	6.1	Reference	
≥60	4.3	1.111 (0.637 to 1.936)	0.711
**Gender**			
Male	5.3	Reference	
Female	6.1	0.900 (0.549 to 1.475)	0.676
**ECOG PS**			
0	6.3	Reference	
1-2	1.7	3.624 (1.750 to 7.505)	**0.001**
**LDH**			
Normal	5.9	Reference	
Elevated	3.2	1.135 (0.592 to 2.176)	0.702
**Subtype**			
Cutaneous	6.1	Reference	
Acral	8.8	0.857 (0.494 to 1.489)	0.585
Mucosal	3.5	1.623 (0.829 to 3.180)	0.158
**Tumor stage**			
Stage III	10.3	Reference	
Stage IV	5.3	1.143 (0.415 to 3.149)	0.796
**Liver metastasis**			
No	8.8	Reference	
Yes	1.9	3.081 (1.645 to 5.772)	**<0.001**
**Brain metastasis**			
No	5.9	Reference	
Yes	2.8	1.714 (0.814 to 3.608)	0.156
**Line of systematic treatment**			
First	6.1	Reference	
Second	3.6	1.046 (0.593 to 1.843)	0.877
**Type of immunotherapy**			
Anti-PD-1/PD-L1/CTLA4 monotherapy	3.4	Reference	
Anti-PD-1/PD-L1+Anti-CTLA-4 therapy	20.3	0.565 (0.230 to 1.385)	0.212
Anti-PD-1/PD-L1+ other therapies	5.9	0.899 (0.532 to 1.519)	0.691

*P values <0.05 in bold are statistically significant.

HHLA2, human endogenous retrovirus-H long terminal repeat-associating protein 2; ECOG PS, Eastern Cooperative Oncology Group Performance Status; LDH, lactate dehydrogenases; PD-1, programmed cell death protein-1; PD-L1, programmed death 1 ligand 1; CTLA-4, cytotoxic T-lymphocyte-associated protein 4.

**Table 3:** Univariate analysis of prognostic factors correlated with OS.

Characteristics	mOS (months)	HR (95%CI)	P value
**HHLA2 expression**			
Low	14.2	Reference	
High	52.4	0.290 (0.129 to 0.653)	**0.003**
**Age**			
<60	17.0	Reference	
≥60	21.5	1.125 (0.619 to 2.046)	0.699
**Gender**			
Male	21.0	Reference	
Female	17.0	0.881 (0.512 to 1.518)	0.649
**ECOG PS**			
0	22.3	Reference	
1-2	8.3	4.467 (2.056 to 9.708)	**<0.001**
**LDH**			
Normal	20.7	Reference	
Elevated	14.0	1.494 (0.769 to 2.902)	0.236
**Subtype**			
Cutaneous	14.4	Reference	
Acral	29.3	0.749 (0.407 to 1.379)	0.353
Mucosal	8.9	1.716 (0.843 to 3.492)	0.137
**Tumor stage**			
Stage III	19.3	Reference	
Stage IV	17.9	1.903 (0.463 to 7.820)	0.372
**Liver metastasis**			
No	23.9	Reference	
Yes	4.8	3.918 (2.059 to 7.455)	**<0.001**
**Brain metastasis**			
No	20.7	Reference	
Yes	12.4	1.401 (0.599 to 3.280)	0.437
**Line of systematic treatment**			
First	16.4	Reference	
Second	17.9	1.270 (0.705 to 2.288)	0.426
**Type of immunotherapy**			
Anti-PD-1/PD-L1/CTLA4 monotherapy	14.2	Reference	
Anti-PD-1/PD-L1+ Anti-CTLA-4 therapy	38.2	0.328 (0.098 to 1.094)	0.070
Anti-PD-1/PD-L1+ other therapies	17.0	0.822 (0.472 to 1.433)	0.490

*P values <0.05 in bold are statistically significant.

HHLA2, human endogenous retrovirus-H long terminal repeat-associating protein 2; ECOG PS, Eastern Cooperative Oncology Group Performance Status; LDH, lactate dehydrogenases; PD-1, programmed cell death protein-1; PD-L1, programmed death 1 ligand 1; CTLA-4, cytotoxic T-lymphocyte-associated protein 4.

In addition, multivariate Cox proportional hazard regression analyses further determined HHLA2 expression as an independent predictor of melanoma survival (PFS: HR=0.268, 95%CI: 0.126 to 0.571, p=0.001; OS: HR=0.334, 95%CI: 0.147 to 0.755, p=0.008, **Table 4**). These results all together indicated the robust value of HHLA2 in predicting prognosis of immunotherapy on melanoma, and it could serve as an ideal biomarker.

**Table:** Table 4 Multivariate analyses of prognostic factors correlated with PFS and OS.

Characteristics	PFS		OS	
	HR (95%CI)	P value	HR (95%CI)	P value
**HHLA2 expression (Low vs high)**	0.268 (0.126 to 0.571)	**0.001**	0.334 (0.147 to 0.755)	**0.008**
**ECOG PS (0 vs 1-2)**	3.041 (1.467 to 6.304)	**0.003**	3.353 (1.527 to 7.361)	**0.003**
**Liver metastasis (no vs yes)**	2.368 (1.261 to 4.447)	**0.007**	2.847 (1.483 to 5.465)	**0.002**

*P values <0.05 in bold are statistically significant.

HHLA2, human endogenous retrovirus-H long terminal repeat-associating protein 2; ECOG PS, Eastern Cooperative Oncology Group Performance Status.

### Relationship between HHLA2 expression and the infiltration of CD8^+^ TILs in melanoma

**Figure 6:**
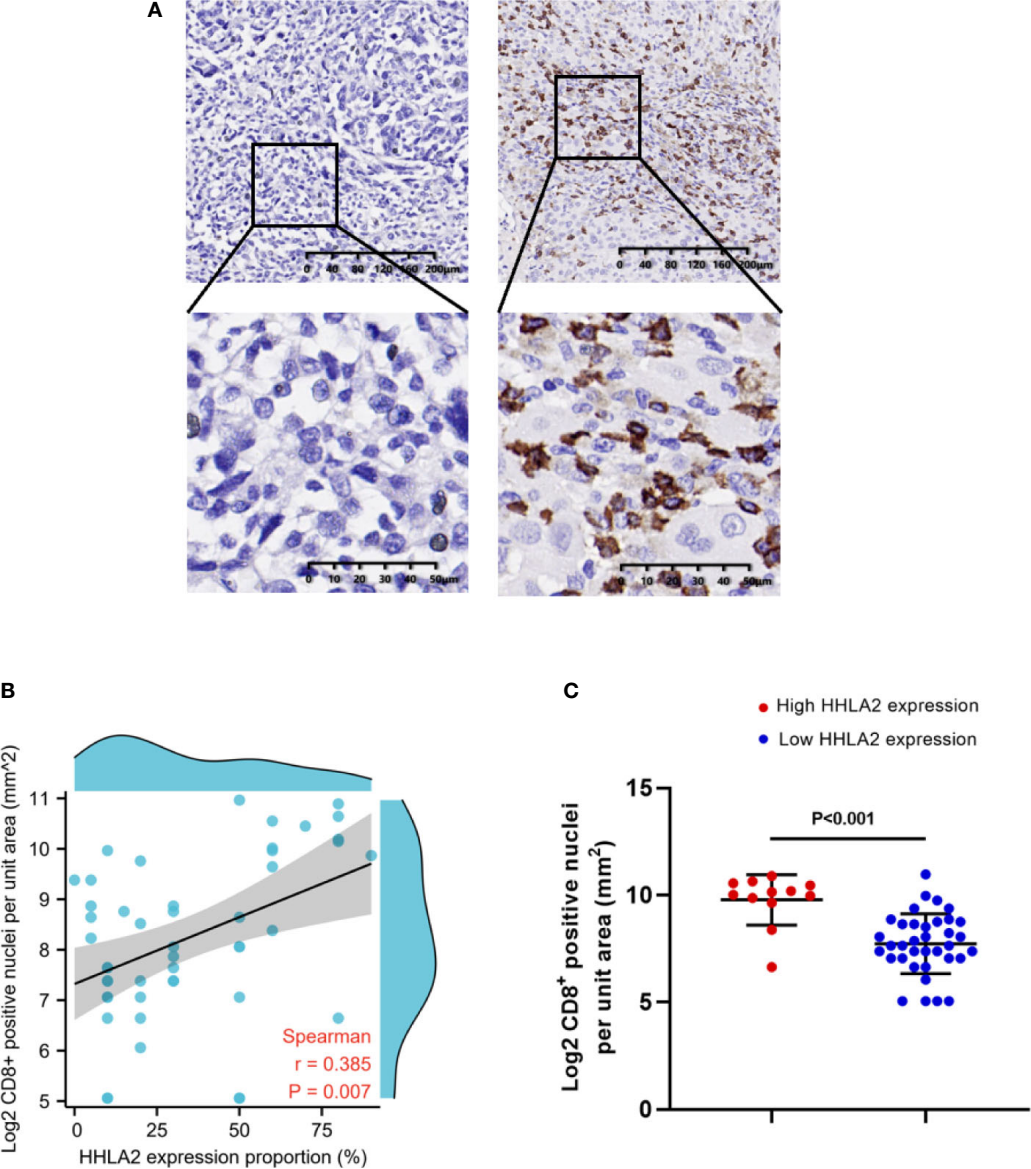
The association between tumor infiltrating CD8^+^ T cells and HHLA2 expression. **(A)** Representative micrographs of CD8 expression and the corresponding negative controls in tumors. **(B)** The association between HHLA2 expression and CD8^+^ T cells (Spearman r=0.385, p=0.007). **(C)** Higher HHLA2 expression was significantly correlated with more intra-tumor CD8^+^ T cells (unparalleled t-test, p<0.001). P values <0.05 are in bold.

CD8^+^ TILs are important immune effector cells in the progress of immunotherapy. High infiltration levels of CD8^+^ T cells have been proved to contribute to prolonged survival of patients after immunotherapy. Hence, the infiltration density of CD8^+^ TILs in FFPE tissues from 49 patients in our cohort was detected by IHC. The results showed that the expression of HHLA2 was positively correlated with the density of CD8^+^ TILs, which was consistent with the results of SKCM microenvironment, as shown in **Figures 6A**, **B** (Spearman r=0.385, p=0.007). The density of CD8^+^ TILs in the HHLA2-high tumors were significantly higher than that in HHLA2-low tumors (p<0.001; **Figure 6C**).

## Discussion

In conclusion, we applied immunohistochemistry to analyze HHLA2 expression pattern in melanoma, and evaluated the influence of HHLA2 expression levels on the efficacy of immunotherapy in ICB-treated patients with advanced or unresectable melanoma. We found that HHLA2 is ubiquitously expressed in tumor cells. Further analyses showed that high HHLA2 expression was correlated with better immune response. Patients with high HHLA2 expression achieved 50% ORR, which was superior to patients with low HHLA2 expression who only achieved 14.3% ORR. Improved ORR also led to better mPFS (31.7 months) and mOS (52.4 months). Analytic data of immune microenvironment showed that the expression level of HHLA2 was positively correlated to the density of CD8^+^ T cells in the tumor microenvironment.

HHLA2 is a newly discovered B7 family molecule, the interactions between B7 molecules and CD28-family receptors are crucial in cancer immunity, the aberrant expression of co-inhibitory B7 molecules has been attributed to reduced anti-tumor immunity and cancer immune evasion. However, the effect of B7-CD28 family on immunotherapy in melanoma is still not very clear. Our study for the first time revealed the potential predictive function of HHLA2 expression pattern on the prognosis of melanoma patients who accept immunotherapy. The expression of HHLA2 in each tumor was analyzed by using TCGA database. HHLA2 is highly expressed in many types of tumors. Although the expression of HHLA2 in melanoma is not as high as other tumors, it is high enough to be distinguished from normal tissues. Through prognostic analysis, we found that high HHLA2 expression was associated with better OS of KIRC, READ, SKCM, and THYM, and worse OS of HNSC, KICH, LIHC, and PAAD. Similar results were confirmed on the issue of PFS, where high HHLA2 expression was associated with better PFS of BRCA, COAD, ESCA, READ, KIRC, LGG, LUAD, PCPG and SKCM, and worse PFS of KICH and THYM. These results were consistent with previously reported points that HHLA2 could be used as an independent prognostic factor for renal clear cell carcinoma and intrahepatic cholangiocarcinoma (25), and high expression of HHLA2 was associated with better prognosis in pancreatic ductal adenocarcinoma (19). Therefore, HHLA2 could be applied as an effective prognostic factor with diversified prognostic impacts according to tumor types.

The different predictive roles of HHLA2 in different tumors may be due to its dual roles in immune response. HHLA2 enhances or inhibits the function of immune cells. TMIGD2, the first identified HHLA2 receptor, is mainly expressed in naive T and NK cells and is quickly reduced upon activation. TMIGD2 stimulates the activity of both T and NK cells (22, 23). KIR3DL3, as the second receptor of HHLA2, is mainly expressed in terminal differentiation effector memory CD8^+^ T cells and CD56^dim^ CD16^+^ NK cells, which mediates co-inhibition of CD8^+^ T cells and NK cells and induces HHLA2^+^ tumor tolerance against tumor killing (24). Therefore, the co-stimulating receptor TMIGD2 and co-inhibitory receptor KIR3DL3 lead to the distinct immune responses of HHLA2.

Through GO analysis and KEGG analysis, we found that HHLA2 played an important role in the immune regulation of melanoma. HALLMARK pathway analysis confirmed that HHLA2 expression was positively correlated with three important immune activation pathways, including IL2-STAT5 signaling pathway, interferon - γ response and interferon - α response pathway. Therefore, HHLA2 may work as an essential co-stimulative molecule in melanoma immune microenvironment.

There were also contradictory results between HHLA2 expression and immune cell infiltration in different cancers. Previously reported studies have proved that high HHLA2 expression was associated with high TIL infiltration in NSCLC (26) and ccRCC (25). Paradoxically, high HHLA2 expression in ICC was associated with low TIL infiltration (15), and there was no clear association in sarcomas (17). It also has been reported that HHLA2 expressed in macrophages was associated with poor survival in patients with hepatocellular carcinoma (27). We firstly used CIBERSORT to analyze the immune infiltration of melanoma patients in the TCGA database. High expression of HHLA2 was associated with High infiltration status of various immune-active cells, including memory B cells, plasma cells, CD8 + T cells, CD4 memory-activated T cells and M1 macrophages. While low expression of HHLA2 was related to the increase of immunosuppressive cell infiltration such as M2 macrophages. To further confirm our results, the infiltration of CD8^+^ T cells in our patient specimens was detected by immunohistochemistry, and the relationship between the infiltration of CD8^+^ T cells and HHLA2 expression was evaluated and analyzed. Consistent with our previous analysis, the density of CD8 ^+^ T cells was positively correlated with HHLA2 expression (p=0.007). Since not all patients were tested for CD8^+^ T cell density in tissue samples, the evidence was not sufficient enough to determine whether CD8^+^ T cells could be used as an independent prognostic factor in our cohort. But it was assured that high CD8^+^ T cell density was associated with high HHLA2 expression. In general, high HHLA2 expression was associated with an immune-activated microenvironment characterized by increased infiltration of CD8^+^ T cells in melanoma.

All these findings suggested that HHLA2 played a positive role in the immune microenvironment of melanoma, which explained for our results. In our study, high HHLA2 expression represented an improved response of immunotherapy, and was significantly and consistently associated with a better PFS and OS of melanoma immunotherapy. We also found that HHLA2 was not associated with any other clinicopathological features, and it was an independent prognostic factor of immunotherapy in melanoma. High expression of immune checkpoint molecules is generally associated with worse prognosis, while high expression of co-stimulative molecules is associated with better prognosis. Our finding further confirmed that HHLA2 served as an important co-stimulative molecule in melanoma, and was necessary for the activation of tumor immune microenvironment.

### Limitation

Several limitations of the study were highlighted as follows. Firstly, the cohort was from a single institution in China, and the expression pattern of HHLA2 in melanoma patients from other institutions is required to be studied. Secondly, due to the lack of patient tissue, only the majority of samples were detected of CD8 positive T cell infiltration. Therefore, it is necessary to use another melanoma cohort to re-confirm the relationship between HHLA2 and CD8 positive T cell infiltration and other related immune cell infiltration, which can also help to understand the role of HHLA2 as a co-stimulative molecule in melanoma.

## Conclusion

In summary, our findings presented an association between HHLA2 expression and the response to immunotherapy in melanoma. High HHLA2 expression predicted better response to ICB, and it was intimately associated with prolonged PFS and OS after immunotherapy and accompanied with increased infiltration of CD8^+^ T cells. HHLA2 may be a potential robust biomarker for identifying patients who may benefit from immunotherapy.

## Data availability statement

Publicly available datasets were analyzed in this study. This data can be found here: 10.6084/m9.figshare.19397093.

## Ethics statement

The studies involving human participants were reviewed and approved by The Ethics Committee of Sun Yat-Sen University Cancer Center. The patients/participants provided their written informed consent to participate in this study.

## Author contributions

All authors participated in data acquisition. F-XH, J-WW, X-QC, X-SZ, YD and D-DL contributed to the conception and design of the study. F-XH, J-WW, J-JL, X-ZW, QZ and J-HW and performed the data analysis and interpretation. F-XH, J-WW, HJ, Q-YD, X-SZ and D-DL contributed to drafting and revision of the manuscript. All authors contributed to the article and approved the submitted version

## Funding

This study received support from the National Natural Science Foundation of China (Grant No. 81772910, 81572493, 81802725, 82002898), Foundation of Sun Yat-sen University Cancer Center for Distinguished Young Scholar (Grant No. PT04180201), General project of Natural Science Foundation of Guangdong Province (2019A1515011188).

## Conflict of interest

The authors declare that the research was conducted in the absence of any commercial or financial relationships that could be construed as a potential conflict of interest.

## Publisher’s note

All claims expressed in this article are solely those of the authors and do not necessarily represent those of their affiliated organizations, or those of the publisher, the editors and the reviewers. Any product that may be evaluated in this article, or claim that may be made by its manufacturer, is not guaranteed or endorsed by the publisher.
