# Supplementary material for: HHLA2 predicts improved prognosis of anti-PD-1/PD-L1 immunotherapy in patients with melanoma
Source: Front Immunol. 2022 Aug 8;13:902167. doi: 10.3389/fimmu.2022.902167 (PMC9395140; doi:10.3389/fimmu.2022.902167)
Supplement: Supplementary file 1 [file DataSheet_1.pdf]

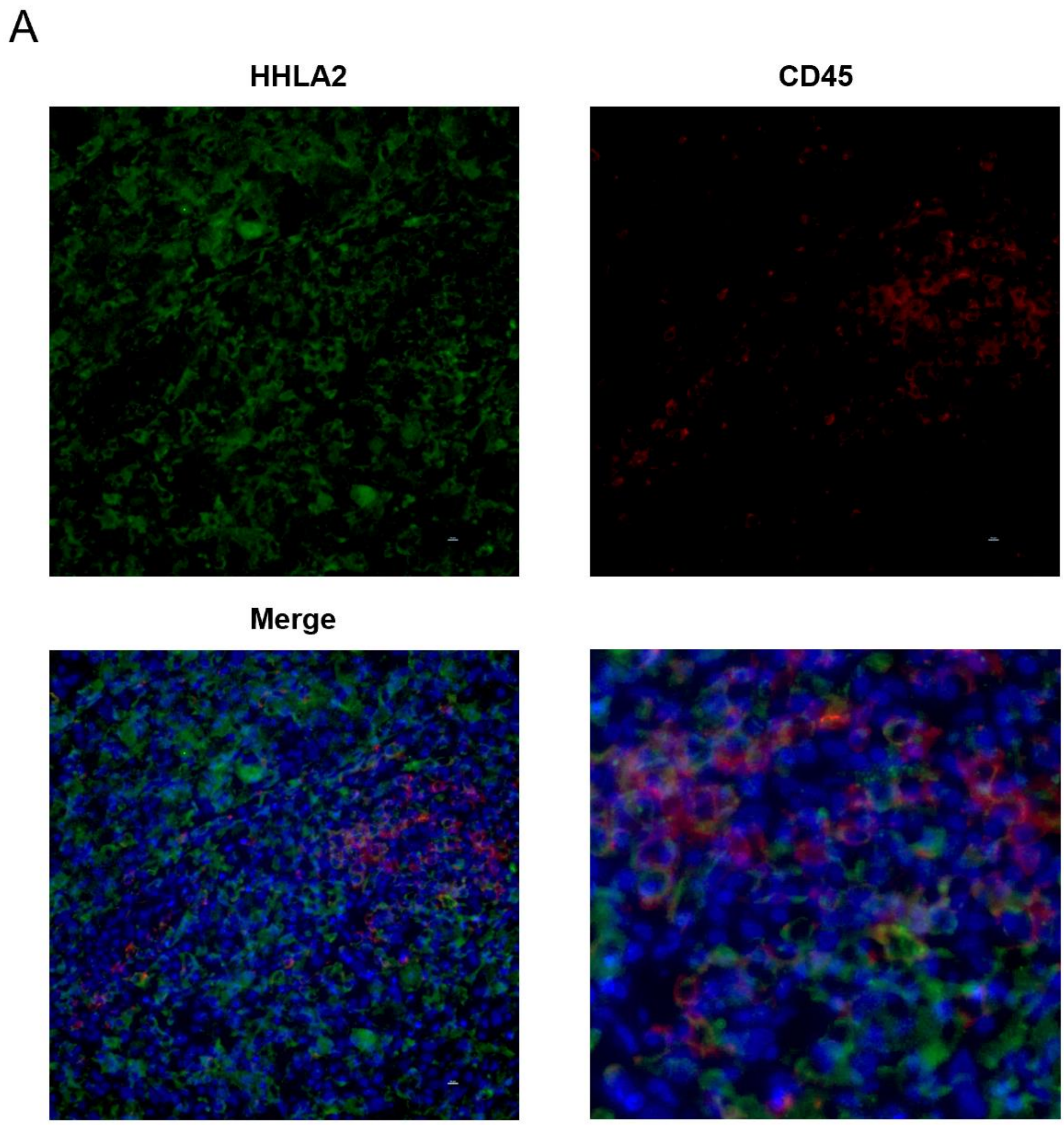

FIGURE S1. (A) representative images of colocalization experiments by immunofluorescence in melanoma tissues. Red, CD45, green, HHLA2; blue, DAPI (4′,6-diamidino-2-phenylindole).

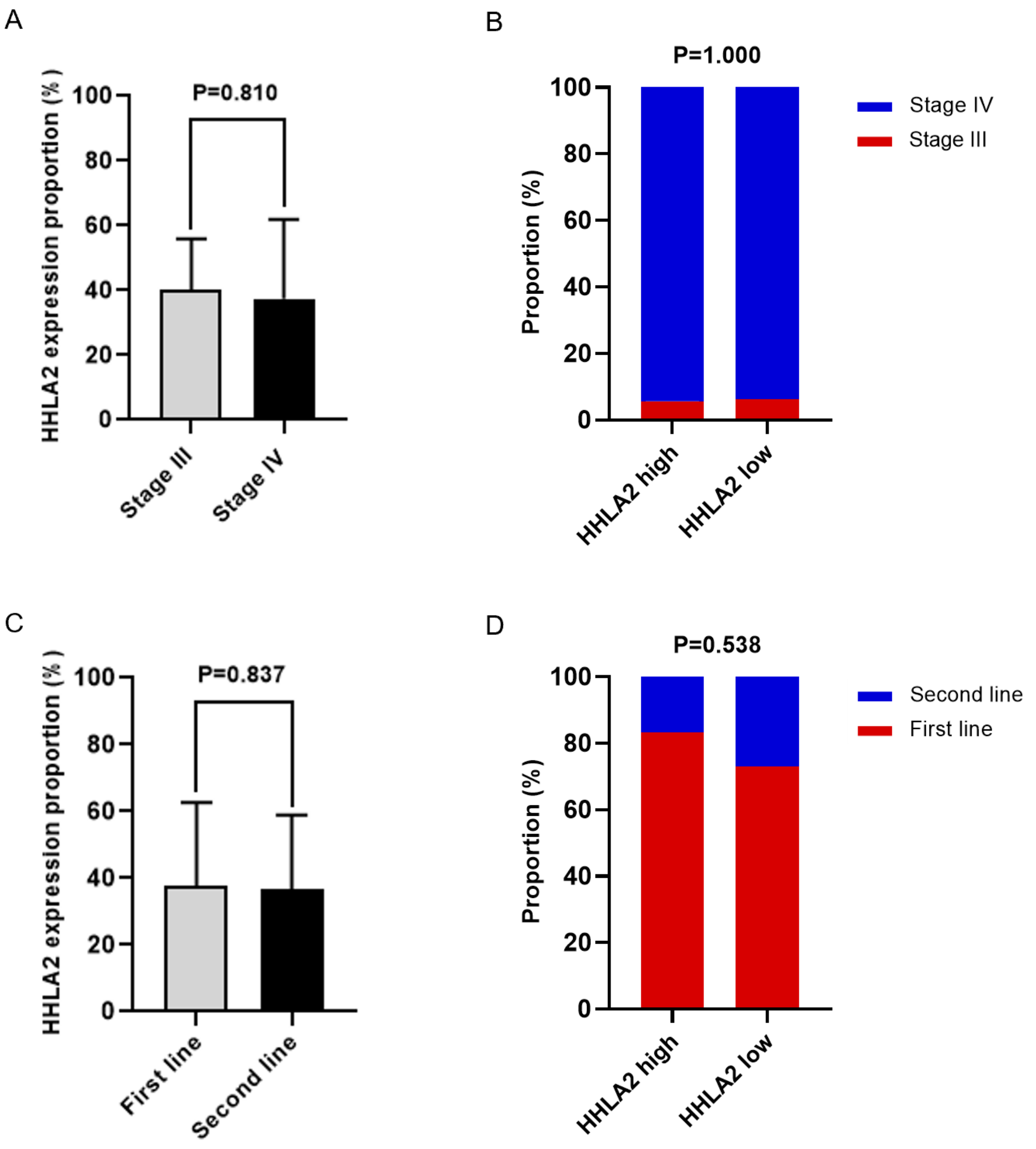

FIGURE S2. (A) The HHLA2 expression proportion within the tumors of stage III and stage IV patients treated with ICB. (B) The distribution of different stages within the tumor of high and low HHLA2 expression group treated with ICB. (C) The HHLA2 expression proportion within the tumors of first line and second line patients treated with ICB. (D) The distribution of different treatment lines within the tumor of high and low HHLA2 expression group treated with ICB.

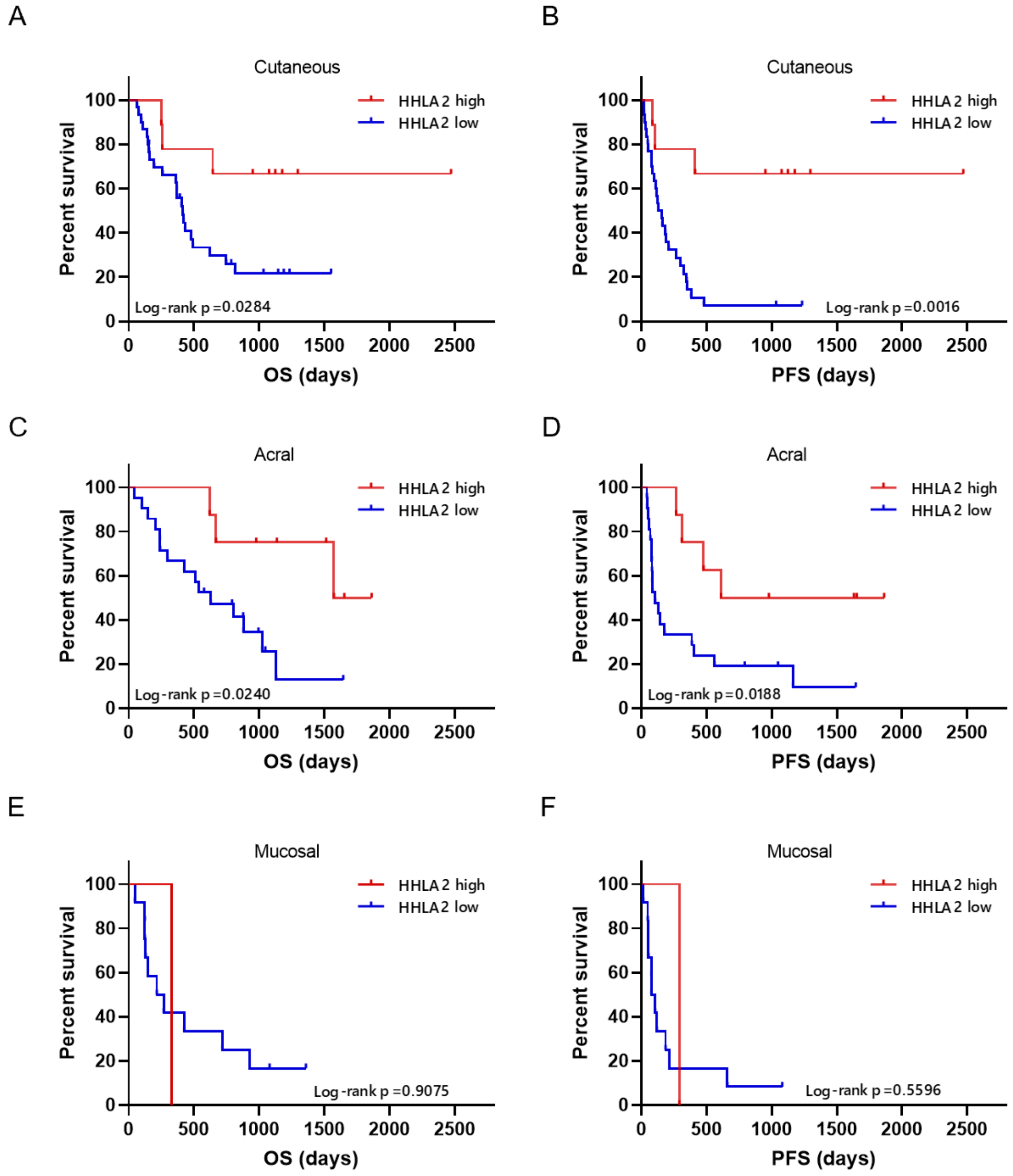

FIGURE S3. (A-B) KM curves showing the association between high HHLA2 expression and improved PFS and OS after treatment with ICB in cutaneous melanoma. (C-D) KM curves showing the association between high HHLA2 expression and improved PFS and OS after treatment with ICB in acral melanoma. (E-F) KM curves showing the association between high HHLA2 expression and improved PFS and OS after treatment with ICB in mucosal melanoma.
